# Discovery of the fish host of the ‘planktonic’ caligid *Caligus undulatus* Shen & Li, 1959 (Crustacea: Copepoda: Siphonostomatoida)

**DOI:** 10.3897/BDJ.8.e52271

**Published:** 2020-06-08

**Authors:** Susumu Ohtsuka, Masaki Nawata, Yusuke Nishida, Masato Nitta, Katsushi Hirano, Kenta Adachi, Yusuke Kondo, Balu Alagar Venmathi Maran, Eduardo Suárez-Morales

**Affiliations:** 1 Fisheries Science Laboratory, Setouchi Field Science Center, Graduate School of Integrated Sciences for Life, Hiroshima University, Takehara, Japan Fisheries Science Laboratory, Setouchi Field Science Center, Graduate School of Integrated Sciences for Life, Hiroshima University Takehara Japan; 2 Fisheries Science Laboratory, Setouchi Field Science Center, School of Applied Biological Science, Hiroshima University, Takehara, Japan Fisheries Science Laboratory, Setouchi Field Science Center, School of Applied Biological Science, Hiroshima University Takehara Japan; 3 Graduate School of Science, Kobe University, Kobe, Japan Graduate School of Science, Kobe University Kobe Japan; 4 Endangered Marine Species Research Unit, Borneo Marine Research Institute, Universiti Malaysia Sabah, Kota Kinabalu, Malaysia Endangered Marine Species Research Unit, Borneo Marine Research Institute, Universiti Malaysia Sabah Kota Kinabalu Malaysia; 5 El Colegio de la Frontera Sur (ECOSUR), Unidad Chetumal, Chetumal, Mexico El Colegio de la Frontera Sur (ECOSUR), Unidad Chetumal Chetumal Mexico

**Keywords:** antagonism, *Caligus undulatus*, life cycle, plankton, *Sardinella zunasi*

## Abstract

The siphonostomatoid copepod *Caligus undulatus* Shen & Li, 1959 has been widely reported from plankton samples obtained from neritic and oceanic waters off coasts of the Indo-West Pacific and Atlantic Oceans. Until now, its fish host has remained unknown. This copepod belongs to an intriguing group of congeners that, despite being part of a chiefly parasitic group, are consistently found as zooplankters. Quite unexpectedly, in October 2019, a fish host of *C. undulatus* was discovered in the Seto Inland Sea, Japan—namely, the Japanese sardinella *Sardinella zunasi* (Bleeker, 1854). Both juvenile (chalimus) and adult individuals of this caligid were observed as parasites of the fish host. The discovery suggests that the species has an alternative life cycle as previously proposed for other purportedly ‘planktonic’ congeners and might frequently switch hosts during the adult stage. Thus, the *C. undulatus* group is newly proposed as a species group in the genus, in which five species are known as planktonic. Some hypotheses on the modified life cycle of caligids also briefly discussed.

## Introduction

Sea lice are a group of siphonostomatoid copepods that parasitise marine teleosts and cause serious economic losses in marine aquaculture worldwide (Ho and Lin 2004, Johnson et al. 2004, Costello 2006, Costello 2009, Shinn et al. 2015). The adverse impact of sea lice infestation on globally-farmed salmon was estimated to cost USD 100–480 million annually (Johnson et al. 2004, Shinn et al. 2015). Studies of the life cycles of sea lice are critical in developing control strategies. Only recently, the life cycles of two major groups, *Caligus* Müller, 1785 and *Lepeophtheirus* Nordmann, 1832, have been clearly differentiated: members of *Caligus* undergo two free-swimming naupliar stages, one infective copepodid, four sessile chalimi and the reproductive adult stage; members of *Lepeophtheirus* have two chalimus and two free-living pre-adult stages instead of four chalimi (Ohtsuka et al. 2009, Venmathi Maran et al. 2013, Hamre et al. 2013).

The work of Ohtsuka et al. (2018) revealed more-flexible life-cycle strategies amongst sea lice than expected, thus recognising three basic patterns involving the post-copepodid stages, wherein: (1) all stages remain on the same host fish with reduced host-switching; (2) chalimi (as well as pre-adults, if any) and adults require intermediate and final hosts, respectively; or (3) adults perform frequent host-switching. The first pattern seems to be the most common amongst sea lice, while the second has so far been found exclusively in three species of *Caligus* which are parasitic on farmed fish. Although further studies are needed, this might be a response of the parasites to the specialised farming situation. The third one is known in 13 species of *Caligus* and one species each of *Lepeophtheirus* and *Metacaligus* Thomsen, 1949, but whose host fish are not yet known (Venmathi Maran et al. 2016, Kim et al. 2019, Ohtsuka and Boxshall 2019). This pattern is clearly represented by *Caligus undulatus* Shen & Li, 1959, widely recorded in plankton samples from off the coasts of the Indo-West Pacific (China, Korea, Japan, India) (Shen and Li 1959, Pillai 1966, Venmathi Maran and Ohtsuka 2008, Venmathi Maran et al. 2012a, Venmathi Maran et al. 2012b, Venmathi Maran et al. 2016, Moon and Park 2019) and the western Atlantic (Mexico, Brazil) (Montú 1982, Suárez-Morales et al. 2012a, Suárez-Morales et al. 2012b, Ortega et al. 2017, Kim et al. 2019). However, until now, it has not been identified as a parasite since Shen and Li (1959) originally described it from 23 adult females (some ovigerous) and 32 males collected in plankton from Kiaochow Bay, China.

During our broad studies on the life cycles of sea lice, we finally found the host fish of *C. undulatus* during a survey of the Seto Inland Sea, western Japan, on 21 and 29 October 2019. Pelagic adults of the copepod were also captured from the surface waters on 31 October 2019. We describe features of the parasite infestation on the local population of the fish host *Sardinella zunasi* (Bleeker, 1854) and discuss the relevance of this finding in explaining the life-cycle strategies of this group of parasitic copepods, which are consistently found in plankton samples, yet without evidence of their hosts.

## Materials and methods

The host clupeid fish of *C. undulatus* was captured off Takehara City, Hiroshima Prefecture, western Japan, by fishing on 21 October 2019. Copepods, parasitic on the body surface of the fish, were removed with forceps. Two adult females of the parasitic copepod are deposited at the National Museum of Nature and Science, Tsukuba City, Japan (NSMT-Cr 27496). Additionally, 24 individuals of the host fish *S. zunasi*, captured off Fukuyama City, Hiroshima Prefecture, were purchased at a fish market at Numakuma, on 29 October 2019. The fish were rinsed with tap water which was then filtered through fine mesh (see Madinabeitia and Nagasawa 2013). In addition to the present finding of *C. undulatus* as a parasite on the body surface of the host fish, we succeeded in collecting planktonic adults of the copepod in the vicinity of the same site, on 24 and 31 October 2019, by surface towing a Maruchi-net (three 10-min tows) with a motorboat. All specimens of *C. undulatus* were observed with differential interference microscopy and photographed with a digital camera attached to a microscope to measure body lengths of the copepods. Body length was measured from the anterior margin of the cephalothorax to the posterior end of the caudal ramus, excluding the caudal setae.

## Results and discussion

**Figure 1.**
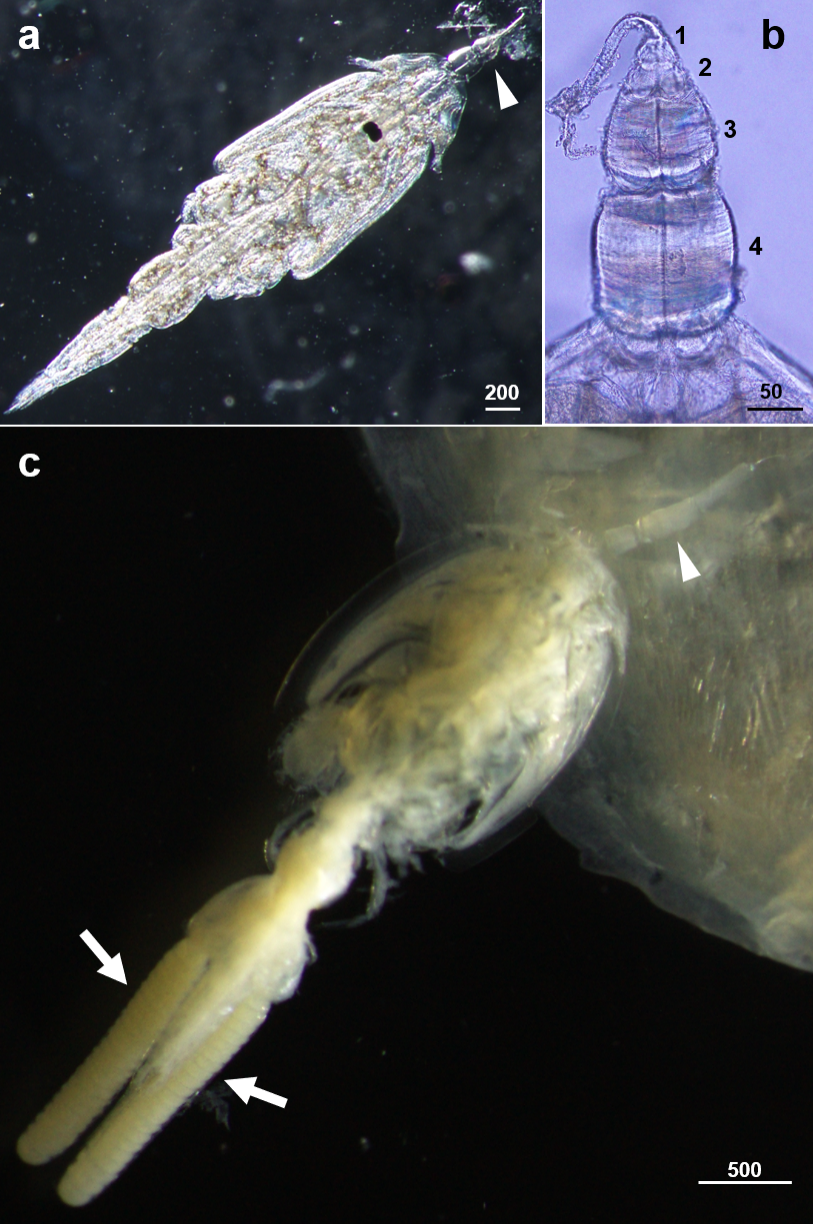
Specimens of *Caligus undulatus* found on the Japanese sardinella *Sardinella zunasi* collected in the Seto Inland Sea, Japan. Scales in μm. **a**: Chalimus IV male (ventral view, before fixation), with the frontal filament indicated by an arrowhead. **b**: Frontal filament of chalimus IV male; extension lobes of copepodid to chalimus IV stages numbered. **c**: Ovigerous adult female (dorsal view, after fixation in 99% ethanol), with the frontal filament and egg strings indicated by an arrowhead and arrows, respectively.

In waters off Takehara City, we discovered the host fish of *C. undulatus* to be the Japanese sardinella *S. zunasi*. In total, four adult females and two chalimi (Fig. 1) were collected from the body surface of six individual fish (each ~150 mm in total length). Body lengths of the adult females ranged between 3.43 and 3.89 mm (average ± standard deviation, 3.70 ± 0.21 mm). The size range of these individuals falls within the previously-known range for *C. undulatus* (3.08–4.46 mm: Moon and Park 2019). Two of the adult females, one of which still had a frontal filament, carried a pair of egg strings (Fig. 1c). The number of eggs per string ranged between 21 and 24, a higher value than was previously reported for the species (4–20: Shen and Li 1959, Venmathi Maran and Ohtsuka 2008, Moon and Park 2019). The present finding of both ovigerous and non-ovigerous adult females on *S. zunasi* suggests that adult *C. undulatus* do not become planktonic immediately after mating on the host. This assumption is supported by the frequent occurrence of adult males of *C. undulatus* in plankton samples, as recorded worldwide (Shen and Li 1959, Pillai 1966, Montú 1982, Venmathi Maran and Ohtsuka 2008, Venmathi Maran et al. 2012b, Moon and Park 2019, present study). Overall, our data show that both mating occurs on the host fish and that adults of both sexes frequently switch host. Two chalimi were tentatively identified as male chalimus III (body length 2.21 mm) and chalimus IV (3.35 mm, Fig. 1) by examination of the number of extension lobes present at the base of the frontal filaments (Fig. 1b) (cf. Piasecki and MacKinnon 1993) and by the presence or absence of lunules under the cuticle of the preceding stage. This is the first report of the chalimi stages of *C. undulatus*. All developmental stages of the species will be described in a future publication.

From the same clupeid fish purchased in Fukuyama City, two ovigerous adult females (egg strings damaged) and seven chalimi of *C. undulatus* were collected from these commercially-captured hosts (97–134 mm in standard length). Two adults were females (body length: 3.81, 3.89 mm) and two male chalimus IV (3.92, 3.34 mm), three male chalimus III (2.17, 2.19, 2.46 mm) and one undifferentiated chalimus II (1.45 mm) stages were collected from hosts. Prevalence could not be calculated, because of detachment of copepods from the hosts during capture and processing of hosts.

In the plankton samples collected off Takehara City, we found a total of 43 adults (13♀, 30♂). The female/male ratio was 0.43 and the frequency of female oviposition was 23.1% (3/13). The number of eggs per egg string ranged between 7 and 16, fewer than in the egg strings carried by the two ovigerous females found on the host *S. zunasi*. Body lengths of females and males were 2.76–3.86 mm (3.32 ± 0.36 mm, n = 13) and 2.46–3.84 mm (3.19 ± 0.36 mm, n = 29), respectively. Since the recorded body lengths are 3.08–4.46 mm for females and 2.82–4.61 mm for males (Moon and Park 2019), the smallest individuals of both sexes were recorded during the present study. Shen and Li (1959) found a sex ratio of 0.88 (28♀♀/32♂♂) and female oviposition frequency of 52.2% (12/23), both higher values than those found in the present study. The temporal occurrence of planktonic adults of *C. undulatus* in waters off Japan, Korea and China appears restricted to August to October (Shen and Li 1959, Venmathi Maran and Ohtsuka 2008, Venmathi Maran et al. 2012a, Venmathi Maran et al. 2012b, Moon and Park 2019); oviposition by planktonic adult females was also recorded in these months (Shen and Li 1959, Venmathi Maran and Ohtsuka 2008, Venmathi Maran et al. 2012b, Moon and Park 2019).

Based on the present and previous studies, it is likely that the life cycle of *C. undulatus* resembles that of other congeners (Ho and Lin 2004, Ohtsuka et al. 2009, Madinabeitia and Nagasawa 2011), though some of its developmental stages remain unknown. The scarce occurrence of adult *C. undulatus* on the host was probably related to frequent host-switching during the adult stage. *Sardinella zunasi* is known to have a restricted distribution along the western coasts of the Japan Sea, East China Sea and northern part of the South China Sea (Froese and Pauly 2019); therefore, infections by the same parasite may also occur in the Chinese and Korean populations of this clupeid. According to Nagasawa et al. (2010), no *Caligus* species has yet been reported as infesting clupeids occurring in Japanese waters. Thus, this is the first record of the occurrence of *Caligus* on a Japanese *Sardinella*, which is a genus of the family Clupeidae, currently containing 22 recognised species and widely distributed in coastal waters of the Indo-Pacific and Atlantic (Froese and Pauly 2019). Especially, *S. aurita* Valenciennes, 1847 and *S. longiceps* Valenciennes, 1847 are abundant in the Atlantic and Indian Oceans, respectively (Chan 1965, Chikhi et al. 1998, Froese and Pauly 2019); accordingly, if *C. undulatus* has a host-specificity to species of *Sardinella*, these are other potential host fishes.

In the speciose genus *Caligus*, six species groups have been previously recognised (Cressey 1991, Boxshall and El-Rashidy 2009, Özak et al. 2017, Boxshall 2018, Ohtsuka and Boxshall 2019). Following Cressey’s criteria separating three species groups, with a 2-segmented exopod of leg 4 defining one species group (Cressey 1991), we propose a newly-recognised species group, the *C. undulatus* group, defined by the following combination of characters (in line with Boxshall 2018): (1) leg 4 3-segmented, with IV spines on the compound distal exopodal segment; (2) three plumose setae on posterior margin of distal exopodal segment of leg 1; (3) outer spines on distal exopodal segment of leg 2 small or reduced; and (4) antenna with weak or well-developed process on proximal segment. In addition, the body is characterised as: (1) having a relatively narrow frontal plate; (2) the female genital complex longer than wide, sometimes with outer margin undulated; and (3) the male urosome slender, with a 2-segmented abdomen. Members of this species group are: *C. undulatus*, *C. evelynae* Suárez-Morales, Camisotti & Martín, 2012, *C. longiramus* Venmathi Maran, Ohtsuka & Jitchum, 2012, *C. ogawai* Venmathi Maran, Ohtsuka & Shang, 2012, *C. tripedalis* Heegaard, 1972 and possibly *C. hyalinae* Heegaard, 1966 (Suárez-Morales et al. 2012a, Venmathi Maran et al. 2012a, Heegaard 1966, Heegaard 1972). Although Heegaard (1966) described a 3-segmented exopod of leg 4 in *C. hyalinae*, the gross morphology of the bodies of both sexes and the antenna and legs 1–3 support its assignment. It is worth noting that these species were all originally described from plankton samples. *Caligus ilhoikimi* Suárez-Morales & Gasca, 2016, described from plankton samples from the Mexican Caribbean, cannot be classified into either the *C. undulatus* group or *C. pseudorhombi* Boxshall, 2018 group, based on the segmentation and armature of leg 4. (see Fig. 3C of Suárez-Morales and Gasca 2016).

As pointed out by Ohtsuka et al. (2018), *C. undulatus* females carry a relatively-small number of eggs per egg string (4–24: Shen and Li 1959, Venmathi Maran and Ohtsuka 2008, Moon and Park 2019, present study) in comparison with those of other congeners, thus suggesting that the species temporarily infests its host to feed and frequently shifts between its hosts and the water column without feeding. The question of why adult *C. undulatus* frequently detach from the host fish at the cost of feeding and reproduction is yet to be answered. The host fish *S. zunasi* is distributed in brackish and coastal waters (e.g. Nakamura 2007, Yoshigou 2015). Some marine copepods, ectoparasitic on euryhaline fish, such as *S. zunasi* and *Lateolabrax japonicus* (Cuvier, 1828), are likely to be lost when they penetrate into fresh waters (Ohtsuka et al. 2007a). It is likely that *C. undulatus* had been detached from the host fish in the fresh or low salinity waters. This hypothesis is supported by evidence that the present planktonic specimens of *C. undulatus* were collected from off the mouth of Kamo River, Takehara City. Actually, freshwater treatment is highly effective at removing sea lice from cultured fish, but the effectiveness depends on the species of sea lice (Ashby 1951, Wootten et al. 1982, Urawa and Kato 1991, Costello 1993, Fajer-Ávila et al. 2008). Another possibility is that there is a kind of evolutionary trade-off between feeding/reproduction and other biological factors (cf. Stearns 1989). In fact, clupeids harbour a wide array of ectoparasitic crustaceans, including copepods and isopods (Williams and Williams 1986, Ho and Lin 2004, Rajkumar et al. 2006, Trilles et al. 2011, Bharadhirajan et al. 2014, Hata et al. 2017, Nagasawa and Isozaki 2017, Rijin et al. 2018). Rijin et al. (2018) found multiple parasitism by isopods or copepods on a single clupeid host and suggested that they exhibit microhabitat segregation for niche separation. The ectoparasitic cymothoid isopod *Anilocra clupei* Williams & Bunkley-Williams, 1986 was reported to parasitise *S. zunasi* in the Seto Inland Sea, but actual data were not given (Williams and Williams 1986). In Thailand and India, the prevalence of other ectoparasitic cymothoid isopods on clupeid hosts ranged from 18% to 54% (Printrakoon and Purivirojkul 2011, Aneesh et al. 2013, Bharadhirajan et al. 2014). A potential antagonistic interaction between these two crustacean ectoparasites on a single clupeid host could promote the temporary presence of adult stage *C. undulatus* in the plankton. This hypothesis is supported by another example of parasite interactions between the dajid isopod *Prodajus curviabdominalis* Simomura, Ohtsuka & Naito, 2005 and the nichothid copepod *Neomysidion rahotsu* Ohtsuka, Boxshall & Harada, 2005, both found inhabiting the marsupium of the mysid *Siriella okadai* collected in the Seto Inland Sea (Ohtsuka et al. 2007). They showed a distinct temporary marsupial habitat segregation, in which the isopod and the copepod mainly occurred at temperatures of > 20°C and < 20°C , respectively (Ohtsuka et al. 2007). Not only multiple endoparasitism (e.g. Sousa 1993, Sandland et al. 2007), but also multiple ectoparasitism (e.g. Fagir et al. 2015) can result in antagonistic interactions. Antagonism could have driven evolutionary shifts in the parasites’ life cycles (Sandland et al. 2007). However, considering the range of egg numbers per egg string and the occurrence of *C. undulatus* adults of both sexes (including ovigerous females), both on the host fish and in the plankton, the duration of adults on the body of, and the timing of their detachment from, the host fish may depend on a combination of biological factors.
